# Association between spinal muscular atrophy type and delayed diagnosis and the risk of spinal deformity in Indonesian patients

**DOI:** 10.1186/s40001-023-01098-3

**Published:** 2023-03-20

**Authors:** Dian Marta Sari, Vitriana Biben, Guswan Wiwaha, Dany Hilmanto

**Affiliations:** 1 Doctoral Study Program, Faculty of Medicine, Universitas Padjadjaran, Bandung, West Java Indonesia; 2 Department of Physical Medicine and Rehabilitation, Faculty of Medicine, Universitas Padjadjaran, Eykman 38, Bandung, 40161 West Java Indonesia; 3 Department of Public Health, Faculty of Medicine, Universitas Padjadjaran, Bandung, West Java Indonesia; 4 Department of Child Health, Faculty of Medicine, Universitas Padjadjaran, Bandung, West Java Indonesia

**Keywords:** Delayed diagnosis, Spinal deformity, Spinal muscular atrophy

## Abstract

**Background:**

Spinal muscular atrophy (SMA) is a genetic disease that causes muscle weakness and atrophy. Delayed diagnosis can lead to loss of motoric functions, which may then progress to deformities such as thoracolumbar scoliosis, pelvic obliquity, and hip subluxation/dislocation. The lack of information or limited experience among healthcare providers and costly genetic tests can cause delayed diagnosis. The current study aimed to assess the characteristics of patients with SMA. Moreover, the association between SMA type and delayed diagnosis and the risk of spinal deformity in the Indonesian SMA Community was evaluated.

**Methods:**

This was a cross-sectional study performed on 53 patients diagnosed with SMA*.* Data about patients’ characteristics were obtained from the Indonesian SMA Community using a questionnaire in August 2019. The information included age, sex, SMA type, age at suspicion and definite diagnosis of SMA, and presence of spinal deformities. Then, descriptive analysis and logistic regression analysis were performed, and the Kruskal–Wallis test and the Chi-square test were utilized.

**Results:**

The median age of patients suspected of SMA was 24 months. A definitive diagnosis of SMA was obtained at 36 months. Further, 43% of patients presented with SMA type 2 and 58% with spinal deformities. Results showed a positive correlation between time interval between suspicion and definite diagnosis of SMA and the risk of spinal deformities (*B* = 0,07; *p* > 0.05). Delayed diagnosis was more common in SMA type 3 than in SMA types 1 and 2, and SMA type 2 was correlated with a twofold risk of spinal deformities (*p* = 0.03; prevalence ratio = 2.09).

**Conclusions:**

SMA type 2 is associated with a twofold risk of spinal deformities. Delayed diagnosis is more common in SMA type 3 than in SMA types 1 and 2. Moreover, there was an association between the time interval between suspicion and definite diagnosis of SMA and the risk of spinal deformities in patients with SMA.

## Background

Spinal muscular atrophy (SMA) is an autosomal recessive genetic disorder characterized by a chronically degenerative alteration in the anterior horn of spinal cord anterior horn. It is caused by a homozygous deletion or mutation in the survival motor neuron (SMN) 1 gene on chromosome 5q13. This gene exists in two forms (a telomeric/SMN1 and centromeric/SMN2) on each allele in humans*.* If the gene does not function appropriately, motor neuronal death and loss of motoric functions can occur, thereby leading to muscle weakness and atrophy and, occasionally, gastrointestinal and respiratory problems [1].

The incidence of SMA is 1 in 6,000–10,000 live births [2]. Moreover, the carrier frequency of SMN1 deletion is between 1/40 and 1/60 in diverse ethnic groups including several East Asian populations [3]. The carrier frequency of Southeast and East Asian populations (1.6–2.1%) was similar to that of Caucasians (2.2%). In contrast, the prevalence of a single SMN1 allele was significantly lower in Black (1%) and Hispanic (1.3%) ethnic groups [4]. 

SMA is classified in types 0, 1, 2, 3, and 4 based on age at which symptoms began and the highest physical milestone achieved. SMA type 0 is extremely severe. The symptoms start before birth, and decreased fetal movement is observed within weeks before delivery. Meanwhile, SMA type 4 causes mild motor impairment, and the symptoms commonly appear in adulthood. Physical abilities can vary per person in each SMA type. In addition, individuals with SMA can lose physical function over time if the muscles continue to weaken. Another SMA classification is based on the highest motor milestone achievement, such as non-sitter, sitter, and walker [5–7].

The gold standard for detecting SMA is the molecular diagnosis of SMN1 homozygous deletion via deoxyribonucleic acid/DNA test, which has a sensitivity rate of 95% regardless of disease severity [5]. However, magnetic resonance imaging and electromyography are occasionally required to determine disease severity [8–10]. However, the high cost of genetic or other supporting tests and the limited resources available can contribute to delayed diagnosis.

The early diagnosis of SMA via newborn screening can facilitate timely intervention, thereby resulting in a better prognosis. Moreover, treatments are currently available. Newborn screening is performed in several countries, including Taiwan [10] and the United States [11]. Moreover, this test is extremely important because early intervention and management before symptomatic treatment can improve motoric development. Therefore, there is an urgent need to implement a diagnostic system because it can allow patients with SMA to undergo early treatment with the potential for maximal therapeutic benefits. These include preventing or slowing the emergence of complications, increasing the chances of survival, and improving quality of life [8, 12].

However, delayed diagnosis can affect disease progression. For instance, it leads to muscle weakness, including respiratory muscles and musculoskeletal issues such as spinal deformities. Severe spinal deformation can cause discomfort, loss of ability to carry out physical activities, and restricted respiratory function. This condition frequently occurs in non-ambulatory patients [13]. Severe scoliosis develops severely in approximately 100% of non-ambulatory patients with SMA [1]. However, no research has assessed the association between delayed diagnosis and spinal deformity progression.

The current research aimed to assess the characteristics of patients with SMA in the Indonesian SMA Community and the association between SMA type and time interval between suspicion and definite diagnosis of SMA and the risk of spinal deformities. Our research may help strengthen the importance of early diagnosis and treatment in SMA, particularly among Indonesians.

## Methods

### Study design

This study was approved by the research ethics committee Universitas Padjadjaran (approval number: 199/UN6.KEP/2020). All participants or their legal representatives provided a written informed consent for the collection and analysis of data for research purposes. All procedures involving human subjects in this study were performed in accordance with the ethical guidelines of the institutional and/or national research committee and the 1964 Declaration of Helsinki.

This was a cross-sectional study using secondary data collected from the Indonesian SMA Community. Data were obtained from a survey conducted by the community using a questionnaire distributed using Google Form® to patients or their caregivers in August 2019. We collected information including age, sex, domicile, type of SMA, age at suspicion and definite diagnosis of SMA, examination test used for diagnosis, posture, orthosis, mobilization device used, type of caregiver, and therapy.

The parameters in the questionnaire were domicile (Java or Non-Java Island), SMA type (1, 2, and 3), SMA diagnostic testing (DNA test or others), body posture (normal or with spinal deformities such as scoliosis, kyphosis, and/or lordosis), orthosis used by the patients (spinal orthosis, others such as ankle–foot orthosis and knee orthosis, combination, or none), mobilization aid (wheelchair, cane, or none), caregiver (family, professional health worker, or both), and therapy (physiotherapy, occupational therapy, both, or none).

### Data analysis

Data about the characteristics of the patients were analyzed via descriptive analysis. Delayed diagnosis was defined as the time interval between suspicion and definite diagnosis of SMA by genetic testing. Moreover, logistic regression analysis was performed to assess the association between the time interval suspicion and definite diagnosis of SMA and the risk of spinal deformity. Moreover, the Kruskal–Wallis test was applied to evaluate the correlation between the type of SMA and delay diagnosis. The Chi-square test was utilized to evaluate the association between SMA type and the risk of spinal deformities. Continuous data were presented as means ± standard deviations or median (minimum–maximum). Meanwhile, categorical data were expressed as frequencies and percentages. The Statistical Package for the Social Sciences software version 25 for Windows (IBM Inc.) was used to analyze data. All statistical tests were performed at a significant level of 5%.

## Results

### Characteristics of the participants

The questionnaire was filled out by 56 respondents from the Indonesian SMA Community. Three respondents refused to participate. Therefore, the participation rate was 94.6%. As shown in Table 1, the number of male participants was greater than that of female patients. The median age of patients with SMA was 82 months. The members of the Indonesian SMA Community mainly lived on Java Island. In this study, to diagnose SMA, DNA testing was performed in 81% of patients.

**Table 1:** Baseline characteristics of patient

Variables	*n* = 53
Age (months), median (min, max)	82 (13, 537)
Sex, *n* (%)	
Male	33 (62)
Female	20 (38)
Domicile, *n* (%)	
Java Island	48 (90)
Non-Java Island	5 (10)
Examination test, *n* (%)	
DNA test	43 (81)
Others	10 (19)
Type of SMA, *n* (%)	
Type 1	16 (30)
Type 2	23 (43)
Type 3	14 (26)
Survival status, *n* (%)	
Alive	47 (89)
Deceased	6 (11)
Tracheostomy, *n* (%)	
Yes	6 (11)
No	47 (89)
Age at SMA suspicion (months), median (min, max)	24 (1, 312)
Age at definite SMA diagnosis (months), median (min, max)	36 (1, 324)
Posture, *n* (%)	
Normal	22 (42)
Spinal deformities	31 (58)
Orthosis, *n* (%)	
Spinal orthosis	6 (11)
Others	11 (21)
Both	6 (11)
None	30 (57)
Mobilization aid used, *n* (%)	
Wheelchair	37 (70)
Cane	1 (2)
None	15 (28)
Caregiver, *n* (%)	
Family	36 (68)
Professional healthcare worker	4 (8)
Both	13 (25)
Therapy, *n* (%)	
Physiotherapy	33 (62)
Occupational therapy	2 (4)
Both	12 (23)
None	6 (11)

*SMA* spinal muscular atrophy

According to SMA type, 30% of patients presented with type 1 SMA, 43% type 2 SMA, and 26% with type 3 SMA. Some members of the Indonesia SMA Community died, and 11% presented with type 1 SMA. In this study, 11% of patients underwent tracheostomy.

The median age of patients suspected of SMA was 24 months, and a definitive diagnosis of SMA was obtained at 36 months. Approximately 42% of patients had normal posture. Meanwhile, 58% presented with spinal deformities. In this study, 22% of patients wore spinal and combination of others orthosis, and 21% used only other devices. About 57% of patients did not wear orthosis (either spinal or others). Moreover, 28% of patients with SMA did not use any aid or devices, 70% utilized wheelchairs and 2% used canes.

In total, 36 (68%) of patients with SMA were cared for by their family, 4 (8%) by health care professionals, and 13 (25%) by both families and health care professionals. In this study, most patients with SMA received physiotherapy (62%), occupational therapy (4%), and both occupational therapy and physiotherapy (23%). However, 11% did not receive any therapy.

This study assessed the association between time interval between suspicion and definite diagnosis of SMA and the risk of spinal deformities in patients with SMA. As shown in Table 2, the coefficient of regression based on crude analysis, there was an association between time interval and the risk of spinal deformities (*B* = 0.007). However, the result was not statistically significant (*p* = 0.446).

**Table 2:** Time interval between suspicion and definite diagnosis of SMA and the risk of spinal deformity

	B	SE	Wald	Crude-OR (95% CI)	*p* value
Time interval	0.007	0.009	0.580	1.007 (0.990–1.024)	0.446
Constant	0.320	0.319	1.004	1.376	0.316

*B* coefficient of regression, *SE* standard error, *OR* odds ratio, *CI* confidence interval

Table 3 shows that patients with SMA type 3 had more delayed diagnosis than those with SMA types 1 and 2. SMA type 1 was diagnosed more quickly than SMA type 2. However, the results did not significantly differ (*p* > 0.05).

**Table 3:** Association between the type of SMA and delayed diagnosis

Delayed timing (months)	SMA			*χ*^2^ (df = 2)	*p* value
	Type 1	Type 2	Type 3		
Median (min–max, IQR)	1 (0–61, 8)	1 (0–84, 6)	12 (0–252, 30)	2.43	0.296^*^

*IQR* interquartile range

^*^Kruskal–Wallis test

Table 4 shows that SMA type 2 was associated with a twofold risk of spinal deformity. Furthermore, there was a significant correlation between SMA type 2 and spinal deformity (*p* < 0.05). The p value for the global test for the association between SMA type and the risk of spinal deformity, as assessed using the Chi-square test, was 0.03.

**Table 4:** Association between SMA type and spinal deformities

SMA	Spinal deformities		Total	PR (95% CI)	*p* value
	Yes	No			
Type 1	6	10	16	Reference	
Type 2	18	5	23	2.09 (1.07 − 4.07)	0.03
Type 3	7	7	14	1.33 (0.59 − 3.03)	

*PR* prevalence ratio, *CI* confidence interval

## Discussion

Indonesia has the fourth largest population worldwide. However, data about the prevalence of SMA and its impact are extremely limited. Our study discussed the characteristics of patients with SMA in Indonesia, and a correlation was observed between SMA type and delayed diagnosis and the risk of spinal deformities in patients with SMA.

Result showed that there was a large disparity in the number of the Indonesian SMA Community members compared to other countries [4, 14]. This might be caused by the lack of knowledge about the signs and symptoms of SMA among parents. As a result, they do not take their child to a hospital with proper facilities, and the child is eventually misdiagnosed [15]. The age of patients with SMA in the community significantly varies. Moreover, the severity and types of SMA remarkably varied as each type has a distinct onset and survival rate [1, 16, 17].

In this research, the patients were commonly men. The sex distribution of patients in this study was in accordance with that in a previous study [18]. SMA is considerably more prevalent in men, with a frequency of almost 60%. Similar findings have been observed in a previous study, which showed that sex may play a role in illness severity. However, the modulation mechanism is not completely known currently [19]. 

Based on the data collected, most members in the Indonesian SMA Community reside in Java Island. This is consistent with the fact that most patients have undergone DNA test for SMA diagnosis. Not all hospitals perform DNA test; thus, not all patients undergo this examination [9]. The low number of patients with SMA in non-Java Island may be attributed to the lack of awareness about SMA disease. Hence, the signs and symptoms are detected at an earlier time and the health services are limited. Health care services, such as diagnostic and imaging equipment, are costly primarily in the major cities of Indonesia and Java Island where Jakarta, which is the capital city, is located [20]. The equipment-to-population ratio in Indonesia is lower than that of most of emerging countries in South-East Asia, such as Malaysia, Singapore, and Thailand [20]. Another obstacle is that Indonesia has the largest archipelago worldwide. Therefore, transportation to other provinces and specific islands are limited. This issue complicates several aspects of health care services, including health promotion and education, diagnostic testing, referral systems, and even therapy [20].

In this study, the number of patients with SMA type 2 was the highest, followed by that of patients with SMA types 1 and 3. This finding was in accordance with that of a study in Spain, which showed that the proportion of patients with SMA type 2 was the highest [21]. Six patients who died were members of the Indonesian SMA Community. All of them presented with SMA type 1, which is the most common and severe type of SMA [1]. SMA type 1 has a higher fatality rate than other types. The life expectancy of individuals with this condition is lower as respiratory failure becomes a significant concern [1]. Approximately 50% of patients diagnosed with SMA type 1 have diaphragm problems, combined with weak intercostal muscles resulting in paradoxical breathing. Aspiration pneumonia is associated with a high rate of morbidity and mortality. Patients with SMA commonly present with a rapid loss of motor and respiratory function during the disease course and within the first year in SMA type 1. However, with noninvasive ventilator assistance, survival beyond 1 year can be increased by more than 70% [1].

Tracheostomy using a ventilator can effectively support patients with SMA who require lifelong and full-time commitment during palliative care since there is still no causal therapy for them. However, in this study, only few patients underwent this procedure during disease progression [1, 16]. Patients with SMA type 2 have a better prognosis, thereby indicating that they have a higher chance of survival and a longer life expectancy.

More than half of the patients had spinal deformities including scoliosis, kyphosis, and lordosis, which can impair posture. Scoliosis occurs almost in all non-ambulant patients with SMA. This is consistent with the result of a previous research showing that patients with SMA type 2 cannot commonly walk [17]. The Wheelchair Provision for Children and Adults with Muscular Dystrophy and other Neuromuscular Conditions guideline have shown that bone curvature begins to occur early in life in young children with conditions such as SMA type 2 or some congenital muscular dystrophy. Spinal curvature was more likely to worsen; thus, patients experience difficulty in sitting. Comorbidities in this population are correlated with orthopedic complications in bone and joint development in progressive scoliosis or joint contractures. If untreated, scoliosis may cause rib-cage deformities leading to respiratory restriction [1].

This study showed that patients with SMA commonly receive rehabilitation therapy. Daily physiotherapy is critical for preventing motor function decline. Although active muscle training can help optimize the remaining muscle force and condition, it is necessary to consider pulmonary limitation and other activity-limiting conditions. Moreover, physiotherapy and respiratory training, along with additional occupational therapy, must be initiated immediately to reintegrate patients into their daily routines and educate them about the use of assistive devices. Maintaining standing ability for at least 2 h per day results in lung function stabilization. Thus, patients should use orthoses and receive standing assistance if necessary. In addition, prompt scoliosis treatment improves lung parenchyma ventilation and stabilizes respiratory function [22].

Half of the respondents in this study wore orthosis, which is supposed to be applied based on the type of SMA. Most respondents used a wheelchair as a mobilization aid. Significant time is spent on a wheelchair. However, patients cannot change their position independently or effectively. This phenomenon was observed in several patients with SMA type 2 who can never walk independently even if they can sit unassisted at some point. Patients with SMA type 3 can independently ambulate. However, some may lose the ability to walk in adulthood due to disease progression [17]. Thus, they need to be supported by a wheelchair. Occasionally, modifications in their environment and home are required to ensure safe access and optimize independence [1]. Hence, patients with SMA required orthosis and other mobilization aid to maintain their ambulation capability and stability during mobilization, decrease musculoskeletal problems such as contracture and scoliosis, and improve posture and respiratory function. Standing abilities should be preserved as much as possible using technical standing and raising devices [22]. In this study, not all patients with SMA received therapy and used orthosis or other mobilization aids. Based on an analysis from in-depth interviews, this finding might be attributed to constraint such as lack of access to health care resources and therapeutic cost, since therapy is not fully covered by insurance, not due to the disease having mild symptoms.

Patients with SMA commonly depend on their caregivers as disability limits the performance of daily activities. In addition, they frequently need assistance in their daily activities, usually from their families or health care professionals. None of the patients in this study performed activities of daily living independently. A previous study in Indonesia also showed that none of the patients practiced self-care, and 74% of patients required partial care and 26% complete care [15]. Moreover, difficulties in living with SMA start from the beginning diagnosis, and the healthcare costs correlated to its treatment, hospitalizations, and consultations also cause a burden. There were three groups of caregivers in this study, and the selection of caregiver is based on the economic condition of the patient's family. A previous study showed that the non-healthcare cost is higher than the healthcare cost in SMA. Non-healthcare costs include informal care provided by non-professionals to patients with SMA to maintain or enhance their autonomy. The number of hospital admissions can determine healthcare cost, drug consumption, emergency visits, outpatient treatment (rehabilitation, medical testing, medical visits, and home medical care), healthcare-related transportation, and the use of assistive devices [21]. This factor should be considered. Thus, more attention can be given to SMA management. Moreover, further research must be conducted to further explore this notion.

This research revealed an association between SMA type and the risk of spinal deformities. SMA type 2 has a twofold risk of spinal deformities. If patients with SMA type 1 survive past infancy and if they are positioned in a semi-upright position, their thorax bends in one direction due to the lack of truncal support. Hence, scoliosis develops [20]. However, in SMA type 1, scoliosis was not a significant issue because severe muscle weakness and the inability to sit independently upright were more evident [17]. SMA type 2 has a better prognosis than SMA type 1. Therefore, the survival rates are also higher since SMA type 1 has a greater mortality rate than SMA type 2 or 3. A better prognosis is associated with a higher survival rate in SMA type 2, thereby making scoliosis progression more noticeable [21].

Further, delayed diagnosis was more common in SMA type 3 than in SMA types 1 and 2. This results was in accordance with that of the study by Lin, which conducted a systematic literature review about delayed diagnosis in SMA [23]. The duration of delay varies according to the severity and type of SMA. The earliest time to diagnosis was observed in patients with SMA type 1 and the longest time to diagnosis in patients with SMA type 3. Hence, the severity of the disease affects time to diagnosis. Patients with SMA type 3 develop late symptoms; thus, the condition is often diagnosed later than SMA type 1 or 2.

This study revealed a positive association between the time interval between suspicion and definite diagnosis of SMA, and the risk of spinal deformities in patients with SMA. Spine deformity is one of the most common complications of SMA. Patients with SMA experience progressive muscle weakness, including muscle weakness in the back that supports the spine, which results in spinal deformity. Spinal deformities cause mobilization and breathing problems [5]. From a clinical perspective, a delayed diagnosis can worsen spinal deformities. However, the results of our study did not significantly differ. Moreover, they are analogous to those of a study conducted in California, which showed a definite delay between symptom onset and diagnosis [23]. Nevertheless, the result did not significantly differ due to the remarkable variations in SMA symptom onset and severity, which can mimic other diseases. Moreover, this may be attributed to the lack of knowledge in this area among several health care practitioners, which results to ruling out alternative diagnosis before considering SMA, and the developmental milestones of children among caregiver and non-coverage of DNA testing by insurance. The speed of diagnosis will affect prognosis, regardless of SMA type.

Early diagnosis and treatment of SMA can also alleviate stress among patients and caregivers. Although the extent of functional loss during delays is unknown, delayed diagnosis may lead to a missed opportunity for appropriate early SMA care. Thus, tools that can facilitate early detection, such as newborn screening, should be required. Prompt diagnosis, particularly in patients with SMA types 1 and 2, will significantly improve the chances of survival and quality of life if optimal care and treatments are provided [23]. 

To date, some drugs have already been introduced and approved for the treatment of SMA by the Food and Drug Administration (FDA). These include nusinersen (SPINRAZA^®^) [24], onasemnogene abeparvovec (ZOLGENSMA^®^) [25], branaplam, and risdiplam, which have significant overall effects if administered early in the disease course [26]. These novel drugs can improve the prognoses and motor functions of infants with SMA [27]. However, they are not currently available in Indonesia and are quite costly. 

An anticipatory action that can be taken is to monitor the growth and development of children. Further, it can help in the early diagnosis of SMA [5]. In addition, caregivers should be educated about the motoric function development of children to assess delays or decreased function and address the limited diagnostic services of SMA. Recently, there has been no specific data about the characteristics or distribution and clinical and socio-economy impact of SMA in Indonesia. Our research can provide potential information to improve management to support quality of life in patients with SMA.

## Conclusions

An association was observed between SMA type and the risk of spinal deformities. Moreover, SMA type 2 was correlated with a twofold risk of spinal deformities. Delayed diagnosis was more common in SMA type 3 than in SMA types 1 and 2. There was an association between time interval between suspicion and definite diagnosis of SMA and the risk of spinal deformities in patients with SMA. However, the result was not statistically significant. In summary, most respondents in this study presented with SMA type 2, used a wheelchair, and received rehabilitation therapy, and they had delayed diagnosis and spinal deformities. 

## Data Availability

The data sets used and/or analyzed in the current study are available from the corresponding author upon reasonable request.

## Abbreviations

DNA: Deoxyribonucleic acid
SMA: Spinal muscular atrophy
SMN: Survival motor neuron
